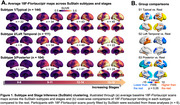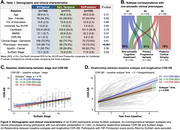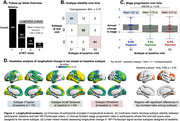# Tau Topography Subtypes in Early‐Onset Alzheimer's Disease: Explaining Clinical Heterogeneity and Propagation Patterns

**DOI:** 10.1002/alz70856_105737

**Published:** 2026-01-08

**Authors:** Marlene Lin, Piyush Maiti, Jiaxiuxiu Zhang, Ganna Blazhenets, Salma Rocha, Ranjani Shankar, Alinda Amuiri, Dustin B. Hammers, Ani Eloyan, Kala Kirby, Robert A. Koeppe, Maria C. Carrillo, Brad C. Dickerson, Liana G. Apostolova, Gil D. Rabinovici, Renaud La Joie, LEADS Consortium

**Affiliations:** ^1^ University of California, San Francisco, San Francisco, CA, USA; ^2^ Memory and Aging Center, Weill Institute for Neurosciences, University of California San Francisco, San Francisco, CA, USA; ^3^ Indiana University School of Medicine, Indianapolis, IN, USA; ^4^ Department of Biostatistics, Brown University, Providence, RI, USA; ^5^ University of Michigan, Ann Arbor, MI, USA; ^6^ Medical & Scientific Relations Division, Alzheimer's Association, Chicago, IL, USA; ^7^ Massachusetts General Hospital and Harvard Medical School, Boston, MA, USA; ^8^ Department of Neurology, Indiana University School of Medicine, Indianapolis, IN, USA; ^9^ Department of Radiology and Biomedical Imaging, University of California San Francisco, San Francisco, CA, USA; ^10^ Global Brain Health Institute, University of California, San Francisco, San Francisco, CA, USA

## Abstract

**Background:**

The clinical heterogeneity of Early‐Onset Alzheimer's Disease (EOAD) is a key factor behind delayed diagnosis within this young(<65yo) group. However, most research has focused on late‐onset amnestic participants and largely underutilized tau‐PET, despite its ability to link neuropathology with clinical outcomes. We aimed to characterize tau‐based subtypes through a robust data‐driven approach in the Longitudinal Early‐onset Alzheimer's Disease Study.

**Method:**

Baseline [18F]Flortaucipir‐PET scans from 365 amyloid‐PET‐positive participants with sporadic EOAD were quantified in 10 regions: left and right medial temporal, lateral temporal, occipital, parietal, and frontal. Tau‐PET values were z‐scored against 85 amyloid‐PET‐negative cognitively normal age‐matched controls and fitted into Subtype and Stage Inference (SuStaIn), an unsupervised clustering algorithm that simultaneously models subtypes and progression from cross‐sectional data.

**Result:**

Three tau‐PET‐based subtypes were identified (Figure 1): Subtype1 (*n* = 144, 39.5%) had a typical bilateral temporoparietal pattern, while Subtype2 (*n* = 111, 30.4%) showed predominant left temporal binding, and Subtype3 (*n* = 104, 28.5%) showed early occipital tau. Subtypes show no significant demographic differences (Figure 2a) but were associated with clinical presentations. Subtype1 was enriched in amnestic participants, while Subtype2 accounted for 61% participants with primary progressive aphasia, and Subtype3 included 79% participants with posterior cortical atrophy (Figure 2b). At baseline, higher SuStaIn stages were associated with higher CDR‐SB (Figure 2c). All subtypes showed longitudinal increase in CDR‐SB, but clinical decline was faster in Subtype1 (Figure 2d). When follow‐up Flortaucipir‐PET scans were fitted to SuStaIn trained on baseline data, 85.6% participants were clustered within the same subtype as their baseline scans; SustaIn stage increased by 0.56/year on average with no difference across subtypes (Figure 3a‐b). Modeling voxelwise tau‐PET over time revealed striking differences (Figure 3c), as each subtype showed significant accumulation in regions that were relatively spared at baseline: tau‐PET increase predominated in the occipital lobe for Subtype1, in bilateral frontal and right temporal areas for Subtype2, and bilateral frontotemporal lobes for Subtype3.

**Conclusion:**

SuStaIn was able to identify robust tau‐PET‐based subtypes that were associated, but not redundant with known clinical phenotypes in AD. Subtypes exhibited differences in prospective clinical decline and patterns of tau‐PET changes, highlighting their potential to refine prognosis and improve progression monitoring in clinical practice and trials.